# OGA: an ontological tool of human phenotypes with genetic associations

**DOI:** 10.1186/1756-0500-6-511

**Published:** 2013-12-05

**Authors:** Jesus Enrique Herrera-Galeano, David L Hirschberg, Vishwesh Mokashi, Jeffrey Solka

**Affiliations:** 1 Genomics and Bioinformatics, Naval Medical Research Center-Frederick, United States Navy, 8400 Research Plaza, Fort Detrick, Frederick, MD, USA; 2 Bioinformatics and Computational Biology, George Mason University, Manassas, VA, USA; 3 Henry M. Jackson Foundation, Bethesda, MDUSA; 4 Center for Infection and Immunity, Columbia University, 8400 Research Plaza, 722 West 168th Street, Room 1704, New York City, NY 10032, USA

**Keywords:** Ontology, Genetic, Association, Structured, Knowledge, Gene, Genotype, Phenotype

## Abstract

**Background:**

The availability of genetic data has increased dramatically in recent years. The greatest value of this data is its potential for personalized medicine. Many new associations are reported every day from Genome Wide Association Studies (GWAS). However, robust, reproducible associations are elusive for some complex diseases. Ontologies present a potential way to distinguish between spurious associations and those with a potential influence on the phenotype. Such an approach would be based on finding associations of the same genetic variant with closely related, but distinct, phenotypes. This approach can be accomplished with a phenotype ontology that also holds genetic association data.

**Results:**

Here, we report a structured knowledge application to navigate and to facilitate the discovery of relationships between different phenotypes and their genetic associations.

**Conclusions:**

OGA allows users to (1) find the intersecting set of genes for phenotypes of interest, (2) find empirical p values for such observations and (3) OGA outperforms similar applications in number of total concepts and genes mapped.

## Background

The impact of genetic associations (GA) is, in some cases, dramatic [1-3]. For example, consider the case of BRACA1/2 [4]. The number of women undergoing a double mastectomy based solely on family history and BRACA1/2 genotype has increased dramatically in the past several years [4]. However, for many phenotypes, especially for complex diseases, this type of association is still elusive. The lack of strong associations after many GWAS studies for phenotypes of interest is known as the “missing heritability” problem [3]. This problem may have its basis in the combinatorial nature of the interaction between genes and phenotypes. Therefore, many efforts have been centered on trying to explore the search space of genes/SNP combinations, or alternatively trying to combine genotypes with expression or epigenetic data [5,6]. Regardless of the approach, all those alternatives tend to ignore the potential *in silico* replication that could be derived from comparing association to phenotypes that are apparently different, but that are indeed related. The basic idea is to take phenotypes that are distinct, but that are related by their ontological relationships, and compare them by finding the genes that are common to them. If common genes are found that have associations with higher frequency than it would be observed by chance, it may indicate that this genotype-phenotype association is stronger than others, or that the genes involved in the association are indeed related to a common root phenotype and not just to their ontological descendants. This type of analysis can facilitate the generation of hypotheses based on this *in silico* replication and can facilitate data management as well as improve data accessibility.

A limited number of applications are currently available that allow linking existing genetic associations to structured knowledge of phenotypes. Two of these applications are Neurocarta [7] and the GWAS Diagram Browser (GDB) [8]. They both allow users to navigate the phenotype ontology and to observe the genetic associations linked to a particular phenotype. However, neither tool allows the user to take advantage of the structured knowledge in the application to test hypotheses or to analyze the data in a way similar to the method proposed above. OGA is different from Neurocarta and GDB in that it allows the user to not only find specific phenotypes and browse the ontology, but it also allows the user to perform meaningful tests that take advantage of the ontology.

## Methods

OGA was created by combining three important data sources: the Human Phenotype Ontology (HPO)[9] to form the backbone of the ontology, the Genetic Association Database (GAD) [10] and the GWASdb[11] to provide the genetic association data. Currently, OGA holds 10,164 ontological concepts imported from HPO, 84,558 genetic associations imported from GAD and 212,185 genetic associations imported from GWASdb. In order to link the concepts in the ontology to the associations obtained from GAD, we utilized an in-house implementation of the suffix array data structure [12]. We created a text index with the HPO concept names and used the index to match the description of the phenotypes in GAD. The mapping between GWASdb and the HPO already exists and it is available at the GWASdb website [13]. We imported the mappings into OGA. OGA is fully implemented in JAVA and runs in all operating systems with the Java Run Environment (JRE). A SQLite database is used to access all data required by the application. Everything necessary to run the application including the jar file, tutorials and the SQLite database is included in a single download file. The only requirement to run OGA is the JRE 1.6 or higher.

## Results and discussion

Currently, OGA has 2,294 (out of 10,164) concepts linked to a total of 135,091 (out of 296,743) associations. A total of 205,707 links now exist in OGA between HPO and the associations in GAD/GWASdb, there are many more links (phenotype to association mappings) than there are associations, because links are many-to-many relationships between associations and phenotypes. The links to several phenotypes in OGA were removed, because the phenotype labels were too short and were causing unspecific matches, i.e. “EO”, “ALS”, “age” and “CAD”. In order to showcase the unique type of analyses that are possible with OGA, we selected four arbitrary neurological disorders: bulimia (HP:0100739), schizophrenia (HP:0100753), depression (HP:0000716) and psychosis (HP:0000709). Four genes common to all four phenotypes were found using OGA: COMT, HTR2A, SLC6A3 and SLC6A4. OGA provides an empirical p value for this observation by obtaining random samples of the same size as the associations established for each phenotype. OGA repeats the random sampling until the same number of genes common to all phenotypes is found by chance (1,000 maximum). For this example, after 1,000 samplings no set was found with four or more genes, which translates into an empirical p value of p < 0.001. Although, they are beyond the scope of this paper, some interesting questions are generated from this *in silico* experiment, such as: what makes these four genes common to all of these phenotypes? Are these simply all associated with a root phenotype such as neurological abnormality (HP:0000707)? Are these genes related to an underlying characteristic common to all four conditions?

OGA allows for easy navigation of the ontology. Additionally, it displays the associations linked to the phenotype selected. Additionally, OGA allows the user to select several phenotypes of interest and to find all the genes common to the selected phenotypes. Furthermore, OGA allows the user to obtain an empirical p value by recreating the random samplings as described above. Although previous work has pointed to the feasibility and benefit of the structured knowledge links between phenotypes and genotypes [14], to our knowledge OGA is the first application to (1) find the intersecting set of genes for phenotypes of interest and (2) find empirical p values for such observations. Two other applications use structured knowledge to relate phenotypes to genetic associations, Neurocarta and the GDB. However, neither application provides a way to take advantage of the ontology by generating analyses based on its structure. OGA provides a way to find the gene intersection between phenotypical hierarchies. When OGA calculates the gene intersection between two concepts this includes the associations to all of the concept's ontological descendants. Neurocarta is manually curated and geared towards neurological diseases, but it does not allow for the type of analysis that OGA provides. Conversely, the manual curation of Neurocarta may make it much more reliable; however it may impair the exploratory potential that can be reached by automated solutions such as OGA. In addition, OGA combines the associations found in GAD and the GWASdb into one application. There are clear advantages of combining both datasets. GWASdb contains many more association entries (241,438) compared to GAD (84,558). GAD, however, contributes a greater overall number of phenotypes to OGA (11,149) compared to phenotypes contributed by GWASdb (1,620). These numbers point to an advantage of OGA versus GDB, OGA contains links to more phenotypes by linking to GAD. It is important to point out that the entries in GWASdb include phenotype-genotype relationships that were established with a p value threshold of p < 10^-3^[11], which is not considered a significant association in the GWAS context (after adjustment for multiple testing) and the entries in GAD are not filtered by association p value. However, this observation benefits the main purpose of OGA, which is to lend weight to unconfirmed associations by finding *in silico* replication in ontologically related phenotypes. Table 1 shows a comparison between OGA, Neurocarta and GDB including several characteristics that help highlight the differences between these tools. By combining GAD and GWASdb entries, OGA reaches 205,707 links (mapping between phenotype concepts and associations) while GDB and Neurocarta only reach 91,301 and 30,000, respectively. Table 1 also shows how the number of concepts with mappings to genetic associations is also higher in OGA, 2,294 compared to 2,000 mapped concepts in Neurocarta and 622 in GDB. The number of genes with mappings to concepts on the ontology is also higher in OGA. OGA has 11,349 genes mapped, while Neurocarta and GDB only reach 7,000 and 7,511, respectively. Table 1 also highlights the point that OGA provides a way to perform statistical analyses, while neither Neurocarta nor GDB have this feature.

**Table 1:** A comparison between OGA, Neurocarta and the GWASdb diagram browser

	**OGA**	Neurocarta*	**GWASdb Diagram Browser**
**Number of links**	205,707	30,000	91,301**
**Number of concepts**	2,294	2,000	622
**Number of genes**	11,349	7,000	7,511
**Backbone**	HPO	HPO, DO, MPO***	EFO
**Curated**	No	Yes	No
**Statistical analysis**	Yes	No	No
**Interface**	Standalone	Website	Website

*The numbers for Neurocarta were taken from [7] **This number was calculated by matching the links from Experimetal Factor Ontology (EFO) to GWASdb (GWAS-EFO-mappings file from [13]). ***Disease Ontology (DO), Mouse Phenotype Ontology (MPO), Human Phenotype Ontology (HPO).

This table shows the comparison between OGA, Neurocarta and GDB for several measures such as the total number of links, concepts and genes.

**Figure 1:**
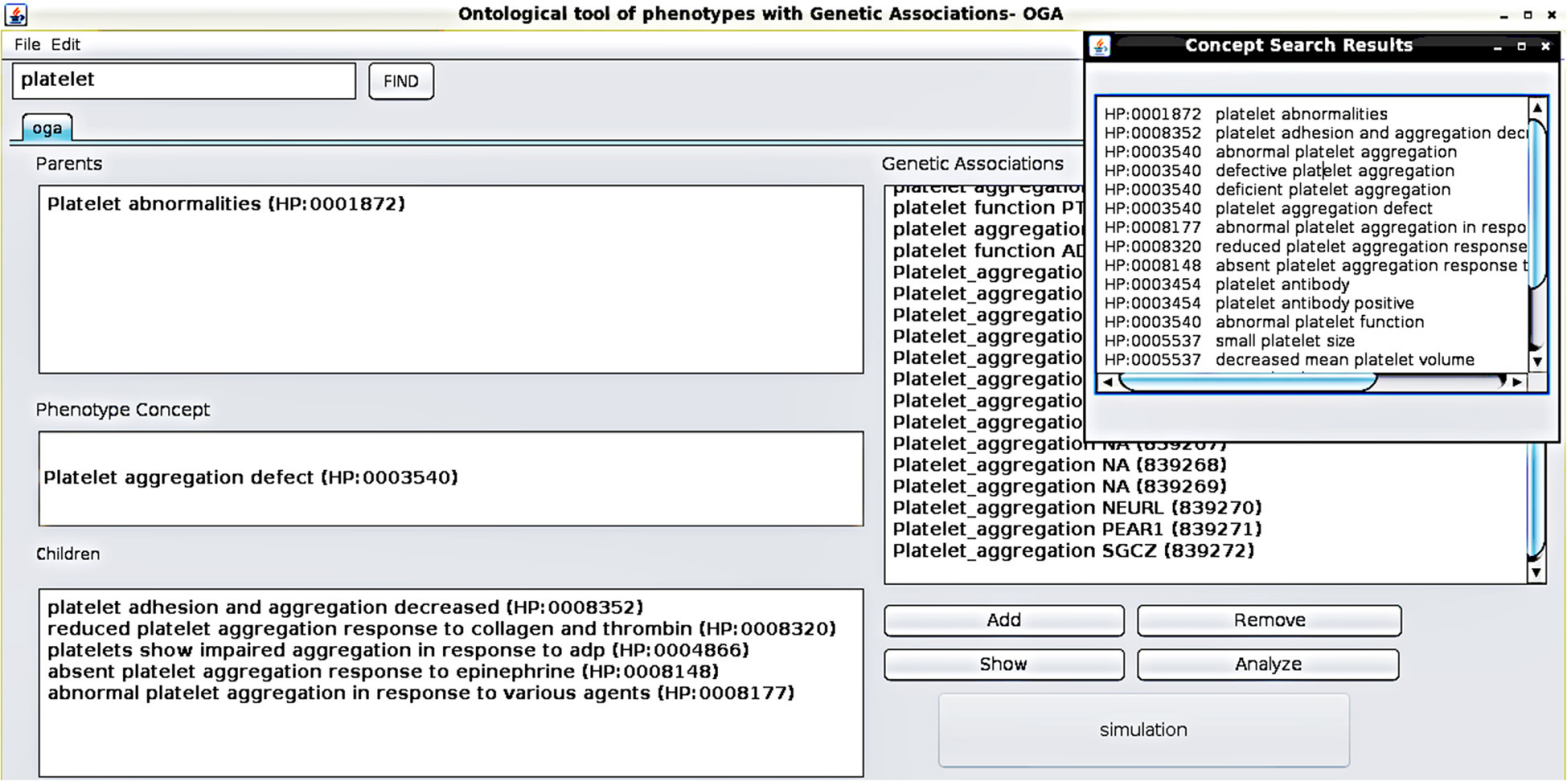
**The OGA graphical user interface.** This figure shows the OGA main window, the search box on the upper right corner can be used to search for any substring of the concept of interest.

OGA is meant to be a very simple to use application, the user can locate any concept present in HPO by typing any substring of the concept in the search box (upper left corner, Figure 1). A list of concepts matching the substring is then presented by OGA (upper right corner, Figure 1). By clicking on the of the search results, the user can navigate to that concept in the ontology. Figure 1 shows how OGA displays the concept selected on the center, in this case, “Platelet aggregation defect (HP:0003540). The concept's ontological parents are displayed right above the concept and its children immediately below (Figure 1). The user can traverse the whole structure of the ontology by simply clicking in the parents or children of a concept or jump to a completely different hierarchy by simply using the search feature. In addition, the user can add the current selected concept to a list of concepts to be analyzed using the “Add” button, then find the gene intersection of the selected concepts using the “Analyze” button (lower right corner, Figure 1).

Finally, OGA is a standalone application that can be extended and used according to the user computational capabilities.

## Conclusions

OGA combines GAD and GWASdb to map genetic associations to the human phenotype ontology. OGA allows users easy navigation of a phenotype ontology that also has links to genetic associations. In addition, OGA can automatically establish links between the concepts present in phenotype ontologies and the entries on genetic association databases. This feature can facilitate the routine reanalysis of data in order to search for emerging associations of interest. Furthermore, OGA provides the means to establish empirical p values for observations of genes overlapping interesting phenotypes. By combining GAD and GWASdb, OGA outperforms similar applications like Neurocarta and GDB in measures such as the total number of mapped concepts, total number of mapped genes and total number of mappings between phenotype concepts and genetic associations.

### Availability of supporting data

**Project name:** Ontological tool of human phenotypes with Genetic Associations (OGA)

**Project home page:**http://code.google.com/p/oga-genetic-association/downloads/list

**Operating system(s):** Platform independent

**Programming language:** Java-Java Run Environment (JRE)

**Other requirements:** none

**License:** GNU GPL

**Any restrictions to use by non-academics:** none

**Contact:** jenriqueherrera@gmail.com

## Abbreviations

OGA: Ontological tool of phenotypes with genetic associations; GAD: Genetic association database; HPO: Human phenotype ontology; GWAS: Genome wide association study; GDB: GWAS diagram browser.

## Competing interests

The authors declare that they have no competing interests.

## Authors’ contributions

JEH, Formulated the questions, designed the application, implemented the applications, wrote the paper. DLH, Participated in the design of the application and provided critical editing of the paper**.** VM, Participated in the design of the application and provided critical editing of the paper**.** JLS, Identifying the problem, design of the application and writing the paper. All authors read and approved the final manuscript.
